# The Use of Intraoperative Neuromonitoring in Lateral Lumbar Interbody Fusion Surgery

**DOI:** 10.7759/cureus.89832

**Published:** 2025-08-11

**Authors:** Rajesh G Arakal, Emily C Courtois, Donna D Ohnmeiss

**Affiliations:** 1 Orthopedic Surgery, Texas Back Institute, Plano, USA; 2 Research, Texas Back Institute, Plano, USA; 3 Research Foundation, Texas Back Institute, Plano, USA

**Keywords:** intraoperative neuromonitoring, lateral lumbar interbody fusion, lumbar spine, neurologic deficit, spine surgery

## Abstract

Introduction: Intraoperative neuromonitoring (IONM) was integrated into most types of spine surgery to help prevent intraoperative nerve injury. Concern has been raised for the actual benefit provided by IONM, particularly in the current era of navigation and robotic guided surgery. The purpose of this study was to investigate IONM changes and postoperative neurologic deficits during lateral lumbar interbody fusion (LLIF).

Methods: This was a cross-sectional study on a consecutive series of 202 patients who underwent LLIF procedures from January 2022 to September 2022. Patients were included if 18 years of age or older and underwent fusion at one, two, or three levels. Data were collected from patient chart review and operative reports, including intraoperative changes in neuromonitoring. Postoperative clinic notes were reviewed for up to approximately three months postoperatively for indication of neurologic injury. IONM was conducted using somatosensory evoked potential (SSEP).

Results: There were two false negatives in this cohort (1.00%), involving no IONM change but exhibiting a neurologic deficit postoperatively. There was one true positive (0.50%) involving a reported IONM change and postoperative neurologic deficit; however, the IONM change occurred just after the patient was flipped prone for the posterior instrumentation portion of the LLIF procedure. The surgeon confirmed through new imaging that the fusion cage was in the appropriate position.

Conclusion: This study found that changes in IONM were rare (0.50%). In the case of the one true positive, this occurred during repositioning, and the IONM change was not successful as a preventative measure. These results indicate that the use of SSEPs may not be supported for the LLIF approach. While medico-legal concerns promote its usage, the low rates of neurologic deficits and costs associated with IONM should be considered, leading to a reexamination of its use in LLIF spinal surgery.

## Introduction

Intraoperative neuromonitoring (IONM) has been used in spine surgery for decades with the purpose of preventing or reducing iatrogenic nerve injury. The greatest risk being addressed was the malpositioning of pedicle screws into the spinal canal or foramen. IONM usage rose 296% between the years 2004 and 2014, and with the increase in spinal surgeries over the years, the use of IONM is also expected to increase [1]. Even with the potential to aid spinal surgeons, there has been increasing debate over the usefulness of IONM due to the rare likelihood of damage to neurological structures, specifically during anterior lumbar spinal approaches [2-4]. These approaches, which can include anterior lumbar interbody fusions (ALIF), oblique lateral interbody fusion (OLIF), and stand-alone lumbar total disc replacements (TDR), pose a smaller neurologic threat than posterior surgical approaches due to the locations of exiting nerve roots from the neural foramen.

In particular, the lateral lumbar interbody fusion (LLIF) (or extreme lateral interbody fusion (XLIF)), which was first recorded in the literature in 2006, consists of working around the psoas muscle, which protects numerous anatomical structures including the obturator and femoral nerves in addition to the vasculature in this area, and is so lateral, that only the L1-L5 discs can be accessed, as the L5-S1 disc space is obstructed by the iliac crest [5]. There is, however, some concern for injury to the lumbar plexus, or network of nerves branching further out from the obturator and femoral nerves in this region. Due to the fine dissection of the musculature in the lateral approach, this network of nerves can remain largely unharmed, although injuries have been known to occur [6,7].

Given the increasing debate surrounding the usefulness of IONM in some spine surgery types, the use of IONM during these procedures has been questioned. The purpose of this study was to investigate the incidence of neurologic deficits after undergoing LLIF surgery to assess whether IONM, particularly the use of somatosensory evoked potential (SSEP), is beneficial for this procedure. 

## Materials and methods

This study was conducted at Texas Back Institute, Plano, Texas, United States. The study was reviewed by the Institutional Review Board of Medical City, Plano, and determined to be exempt (reference number: 2024-416).

Comprehensive surgical records from a multi-site spine specialty institution were obtained for January to September, 2022, to identify a consecutive series of patients who underwent a lateral approach lumbar spine surgery. Patients were included if they (i) had undergone a lateral interbody fusion (LLIF or XLIF), (ii) had surgery performed on one, two, or three lumbar levels, and (iii) were at least 18 years of age at the time of surgery. These lumbar spine surgeries were primarily performed for one or more of the following conditions: symptomatic degenerative stenosis, low-grade spondylolisthesis, and/or disc degeneration unresponsive to non-operative care. Patients were excluded if they underwent surgery for trauma, tumor, or deformity.

If identified for inclusion in the study, the patient’s clinic charts were reviewed for general descriptives (age at the time of surgery, sex, height, weight). Operative notes were reviewed for surgical details, including lumbar levels operated on and IONM changes. If there was a change in IONM, the surgeon’s response was also recorded. The primary endpoint of the study was the occurrence of an iatrogenic neurological deficit, or lack thereof. Clinic charts were then reviewed postoperatively up to three months for indications of new neurologic deficits. Neurologic deficits were defined as any new lower extremity weakness or severe sensory deficits not present or not more severe than pre-operatively. Anticipated changes in sensation, such as thigh tingling or numbness, were not considered sensory deficits. Patients were evaluated during recovery in the hospital for indications of a change in neurological condition and for significant pain onset. If either were present, this was immediately evaluated in detail, including neurological examination.

SSEP was used for every surgical procedure (whether or not additional neurological monitoring was also used). All IONM was performed by an experienced provider specializing in this service. An IONM technician was always present in the operating room while a neurologist monitored the cases from a remote location in real time.

IONM results were classified into three categories of interest: (i) true positive, (ii) false positive, and (iii) false negative. True positive was defined to be a change in IONM in a patient that had a negative change in neurological function postoperatively, or the surgeon altered the surgery intraoperatively to avoid neurological injury. Altering the procedure intraoperatively to avoid or reduce the risk of injury to neural tissue or to avoid re-operation to address iatrogenic neural injury would be considered a beneficial true-positive. A false-positive was defined to be a change in IONM during surgery, but the patient had no new neurological deficit after surgery, nor did the surgeon take action during surgery as a preventative measure. A false negative finding was defined as no change in IONM, but the patient had a demonstrable new neurological deficit after surgery. We did not calculate the rate of true negatives, as this is the expected routine course.

Descriptive data were reported using means for continuous variables, and counts with percentages were calculated for categorical variables. The rates of true positive, false positive, and false negative findings for IONM were calculated as defined above. If an appreciable number of patients had any of these three findings, regression analysis was to be conducted, attempting to identify demographic and/or surgical factors possibly related to it (setting the alpha to 0.05). All data analyses were performed using IBM SPSS Statistics for Windows, version 28.0 (IBM Corp., Armonk, New York, United States).

## Results

A consecutive series of 202 patients were identified. The majority of these patients were female (n=118; 58.4%), with an average age and BMI of 65.3 years and 30.7 kg/m^2^, respectively (Table 1). The majority of the patients underwent a 1-level LLIF procedure (n = 124). All procedures were performed under unconscious sedation using general endotracheal anesthesia. Twenty patients (9.9%) received paralytic anesthesia as this was one surgeon’s preference.

**Table 1:** Characteristics of included patients (N=202)

Characteristics	Values
Age (years), mean±SD	65.3±9.83
BMI (kg/m^2^), mean±SD	30.7±6.44
Female (vs. Male), n (%)	118 (58.4)
Surgery	
Levels Operated, n (%)	
1-level	124 (61.7)
2-level	61 (30.3)
3-level	16 (8.0)

There were two false negatives in this cohort (1.00%), which involved no reported IONM change but exhibited a neurologic deficit postoperatively (Table 2). One of these patients had a reported weakness in the left iliopsoas and sensory deficit in the left quadriceps, but otherwise normal sensation in the lower extremities. The other patient reported left hip flexor weakness and diffuse numbness and tingling, but otherwise intact strength throughout the lower extremities. Both patients showed improvement over the following first postoperative month. In both cases, detailed IONM reports were available in the clinic chart. For the SSEP monitoring, a significant change was defined to be either a minimum 50% decrease in amplitude or a minimum 10% shift in latency. In both cases, EMG was also used as part of the IONM, and no significant change was noted for this modality. Neither of these cases used paralytic anesthesia.

**Table 2:** Patients with neurologic deficits or positive IONM recordings IONM: intraoperative neuromonitoring

Patient ID	Age (years)	Sex	Levels Operated	IONM Recordings	Postoperative Neurologic Deficit
1	80	Male	L4-L5	None	Left hip flexor weakness and some numbness and tingling throughout legs, but otherwise normal sensation in the lower extremities.
2	63	Female	L3-L5	None	2/5 motor strength in left iliopsoas. There is also sensory deficit in the left quad, but otherwise normal sensation in the lower extremities. Improved within 1-month post-op
3	59	Female	L4-L5	Surgeon notified by neuromonitoring of diminished signal in left quadriceps after the anterior portion of the surgery was completed and patient was just positioned prone, but somatosensory are intact and all other motors are intact and at baseline. Confirmed with imaging that everything looks appropriate with regard to the placement of the anterior cage with no encroachment on spinal canal, neural foramen or neurologic structures. Proceeded with the posterior portion of the surgery without any changes.	Significant weakness in left hip flexor and quad. Seen some improvement but still feels numbness in her anterior thigh on the left and weakness especially in hip flexor. Gradually improving.

There was one true positive (0.50%) involving a reported change in IONM with a resultant neurologic deficit postoperatively (Table 2). The IONM change was seen as diminished signal in the left quadriceps and occurred just after the patient was flipped prone for the posterior instrumentation portion of the LLIF procedure. The surgeon confirmed through new imaging that the fusion cage was in the appropriate position, and there was no evidence of encroachment on the spinal canal, neural foramen, or neurologic structures. The surgeon elected to continue with the posterior surgical course and IONM returned to baseline before the completion of the case. Postoperatively, the patient exhibited significant weakness in the left hip flexor and quadriceps as well as numbness in the anterior thigh. The results of this true-positive are complicated by two additional factors involving unique patient anatomy. During the lateral surgical approach, the surgeon dictated the encounter of a unique neurologic structure on top of the disc space field. This nerve was protected and the surgeon avoided manipulating this nerve structure. Paralytic anesthesia was not used in this case.

Of note, there was one case involving a minimal pedicle screw breach into the spinal canal, prompting the surgeon to revise his approach based on additional imaging taken intraoperatively. There was no change in IONM during the entirely of this case and this patient did not have a postoperative neurologic deficit.

There were insufficient numbers of true positive, false positive, or false negative cases to perform a valid regression analysis attempting to identify factors related to these occurrences.

## Discussion

In this large consecutive series of 202 patients who underwent LLIF procedures, changes in IONM were rare (0.5%), and not necessarily related to a postoperative neurologic deficit. In two patients who had a neurologic deficit, there was no reported change in SSEP values. There was one patient who had a change in SSEP values and a noted postoperative neurologic deficit; however, the change occurred while moving the patient, not while the surgeon was operating. This observation prompted the surgeon to continue with the posterior stage of the operation without adjustment to the anterior portion, and the SSEP values returned to baseline before the completion of the case. This patient’s case involved a unique nerve structure, which may have increased the risk of experiencing neurologic deficits. Of note, the use of paralytic anesthesia may render IONM readings inaccurate; however, the majority of the surgeries did not utilize paralytics and false-positives were exhibited in the cases without its use. Out of the 202 cases, there was no case where IONM provided demonstrable benefit to the patient.

Currently, there are no standard guidelines for neuromonitoring usage during spine surgery, and definitive evidence in this area is lacking. A 2021 literature review, by Hofler and Fessler, proposed that different types of IONM can be utilized depending on the type of spine surgery being performed to better monitor potential neurological changes [8]. They suggested electromyography (EMG) would be helpful in lateral approaches to identify neural structures crossing the surgical field, whereas the anterior and posterior approaches would benefit from the use of SSEPs and motor-evoked potentials (MEPs) to assess possible intraoperative nerve injury.

The current study’s reported rate of neurologic deficit (0.5%) is lower than, but also comparable to, other studies using IONM for this procedure, with reports of 0.7% out of 600 XLIF patients, 1.0-2.2% in a meta-analysis of 1409 XLIF patients, and 2.9% of 235 LLIF patients [9-11]. In a meta-analysis involving 6,819 LLIF patients over 11,325 operated levels, postoperative persistent neurologic deficits occurred at a rate of 3.98% [12]. The rates reported after operations using IONM modalities are largely comparable to studies examining deficits without IONM usage, which have been reported at 3.8% and 5% [13,14]. There is data showing a higher incidence of deficits stemming from the lower lumbar levels rather than the more cranial levels, supporting the theory that neurologic injury varies per operated level [9,10]. Based on the literature, the recommendation for IONM usage during lateral approaches is unclear. Interestingly, one study states that the nerves at greatest risk for injury during LLIF surgery are sensory nerves that cannot be monitored in real-time intraoperatively, further questioning its use [11]. A debate on the use of IONM in LLIF procedures demonstrated arguments for both sides, noting that specific changes in the technical surgical approach could influence the rate of deficits, due to the dissection method of the psoas muscle [15]. Additionally, a 2024 study revealed that IONM recording and set-up errors occurred in 5.3% of the examined 454 surgical cases; however, of this study’s total cohort, only seven (1.5%) were lateral approaches and 25 (5.5%) were lateral with either anterior or posterior approaches [16]. The current study’s cohort could not confirm the causes of these occurrences, but recording or setup errors could not be ruled out. To better aid surgeons in IONM usage, a protocol has been described, which has seen a decrease in IONM changes and changes in surgical responses to reduce the incidence of postoperative deficits [17].

While the rates of neurologic deficit seem to vary, there are also cost and medico-legal concerns with routine IONM usage. Of note, some insurance providers consider the use of IONM to not be medically necessary for many spine surgery types [18-20]. It has been reported that using IONM during less complex, single- or two-level spinal procedures may lead to increased healthcare costs without demonstrable benefit; however, its usage in larger multilevel constructs such as deformity cases may decrease long-term healthcare costs [21,22]. Furthermore, a 2021 study examining the use of IONM in posterolateral lumbar fusions (PLF) found that total hospitalization charges were 11% greater for patients with IONM than those without [22]. The authors reported no benefit from the use of IONM in this population. Another study investigated the cost of IONM in minimally invasive transforaminal lumbar interbody fusion versus without IONM [23]. Their data suggested IONM added $4,000 to $5,000 per case. In both groups, slightly more than 5% of patients underwent reoperation for pedicle screw breaches, concluding that the monitoring provided no demonstrable benefit. The cost-effectiveness of IONM may depend on spine surgery type or other unique factors, and it still remains controversial. 

There are also medico-legal concerns that have impacted IONM usage. An outlook on IONM as the legal standard of care for spinal surgeries has led some surgeons to feel pressured to use it regardless of spinal surgery type [24,25]. The opinion that IONM has been overused for spinal surgery tends to apply to non-complex anterior surgeries, such as single-level ALIF or lumbar TDR procedures, whereas deformity surgeries involving significant risk to the spinal cord have maintained strict IONM usage. Beyond spinal surgery type and number of operated levels, which may promote the usage of more safety precautions such as IONM, appropriate patient selection and individual risk factors are also important to consider when defining the most risk-averse course of action.

The current study had the limitations inherent to retrospective designs. Although IONM was used in all cases, in the form of SSEPs, the more specific type of neuromonitoring for each case could not be discerned. Some types of IONM are used for navigation purposes during surgery, while others are used to provide monitoring for the overall surgery, and the surgeons are only notified upon IONM changes. The strengths of this study include the large sample size and the focus on LLIF, or XLIF, operations; this specific criterion allowed an investigation into neurologic injuries related to this operation type.

Given this evidence, the LLIF procedure may not require IONM; however, other procedures still may benefit from its use. There is a need for more studies in this area to determine which spinal surgery operations would benefit from IONM usage. This information may help to provide more guidance for establishing protocols of IONM using in different spine surgery types and patient indications.

## Conclusions

This study found that changes in IONM were rare (0.50%). In the case of the one true positive, this occurred during repositioning, and the IONM change was not successful as a preventative measure. These results indicate that the use of IONM may not be supported for the LLIF spinal procedure.

While there are medico-legal concerns that prompt its usage, the low rates of neurologic deficits and the heightened costs associated with implementing IONM should be considered, leading to a reexamination of its use in the LLIF spinal surgery approach.

## References

[1] Laratta JL, Shillingford JN, Ha A (2018). Utilization of intraoperative neuromonitoring throughout the United States over a recent decade: an analysis of the nationwide inpatient sample. J Spine Surg.

[2] Blumenthal SL, Edionwe JI, Courtois EC, Guyer RD, Satin AM, Ohnmeiss DD (2024). Is the use of intraoperative neuromonitoring justified during lumbar anterior approach surgery?. Int J Spine Surg.

[3] Agarwal N, Nwachuku EL, Mehta A (2020). Perioperative neurological deficits following anterior lumbar interbody fusion: risk factors and clinical impact. Interdisciplinary Neurosurg.

[4] Dowlati E, Alexander H, Voyadzis JM (2020). Vulnerability of the L5 nerve root during anterior lumbar interbody fusion at L5-S1: case series and review of the literature. Neurosurg Focus.

[5] Ozgur BM, Aryan HE, Pimenta L, Taylor WR (2006). Extreme lateral interbody fusion (XLIF): a novel surgical technique for anterior lumbar interbody fusion. Spine J.

[6] Isaacs RE, Hyde J, Goodrich JA, Rodgers WB, Phillips FM (2010). A prospective, nonrandomized, multicenter evaluation of extreme lateral interbody fusion for the treatment of adult degenerative scoliosis: perioperative outcomes and complications. Spine (Phila Pa 1976).

[7] Lykissas MG, Aichmair A, Hughes AP (2014). Nerve injury after lateral lumbar interbody fusion: a review of 919 treated levels with identification of risk factors. Spine J.

[8] Hofler RC, Fessler RG (2021). Intraoperative neuromonitoring and lumbar spinal instrumentation: indications and utility. Neurodiagn J.

[9] Rodgers WB, Gerber EJ, Patterson J (2011). Intraoperative and early postoperative complications in extreme lateral interbody fusion: an analysis of 600 cases. Spine (Phila Pa 1976).

[10] Pumberger M, Hughes AP, Huang RR, Sama AA, Cammisa FP, Girardi FP (2012). Neurologic deficit following lateral lumbar interbody fusion. Eur Spine J.

[11] Palacios P, Palacios I, Palacios A, Gutiérrez JC, Mariscal G, Lorente A (2024). Efficacy and safety of the extreme lateral interbody fusion (XLIF) technique in spine surgery: meta-analysis of 1409 patients. J Clin Med.

[12] Hijji FY, Narain AS, Bohl DD (2017). Lateral lumbar interbody fusion: a systematic review of complication rates. Spine J.

[13] Bobinski L, Liv P, Meyer B, Krieg SM (2023). Lateral interbody fusion without intraoperative neuromonitoring in addition to posterior instrumented fusion in geriatric patients: a single center consecutive series of 108 surgeries. Brain Spine.

[14] Krieg SM, Bobinski L, Albers L, Meyer B (2019). Lateral lumbar interbody fusion without intraoperative neuromonitoring: a single-center consecutive series of 157 surgeries. J Neurosurg Spine.

[15] Cheng I, Acosta F, Chang K, Pham M (2016). Point-counterpoint: the use of neuromonitoring in lateral transpsoas surgery. Spine (Phila Pa 1976).

[16] Mandir A, Ebinger K, DeBruyn L, Kenney K (2024). A prospective look at the prevalence of setup electrode-swap errors across over 450 intraoperative neuromonitoring cases. Neurodiagn J.

[17] Mundis GM, Record NC, Shahidi B (2024). Incidence of postoperative neurological deficit with the use of an intraoperative neuromonitoring protocol for lateral lumbar interbody fusion. J Neurosurg Spine.

[18] (2024). Medical Policy- 7.01.562: Intraoperative Neurophysiologic Monitoring.

[19] Aetna Aetna (2024). Aetna: Intraoperative neurophysiological monitoring. Policy Number: 0697: Intraoperative Neurophysiological Monitoring.

[20] (2022). Medica Coverage Policy: Intraoperative Neurophysiologic Monitoring. Medica Coverage Policy: Intraoperative Neurophysiologic Monitoring.

[21] Nuwer MR, Dawson EG, Carlson LG (1995). Somatosensory evoked potential spinal cord monitoring reduces neurologic deficits after scoliosis surgery: results of a large multicenter survey. Electroencephalogr Clin Neurophysiol.

[22] Austerman RJ, Sulhan S, Steele WJ, Sadrameli SS, Holman PJ, Barber SM (2021). The utility of intraoperative neuromonitoring on simple posterior lumbar fusions-analysis of the National Inpatient Sample. J Spine Surg.

[23] Garces J, Berry JF, Valle-Giler EP, Sulaiman WA (2014). Intraoperative neurophysiological monitoring for minimally invasive 1- and 2-level transforaminal lumbar interbody fusion: does it improve patient outcome?. Ochsner J.

[24] Bible JE, Goss M (2022). To use or not use intraoperative neuromonitoring: utilization of neuromonitoring during spine surgeries and associated conflicts of interest, a cross-sectional survey study. J Am Acad Orthop Surg Glob Res Rev.

[25] Brook M, Irle K (2016). Litigating intraoperative neuromonitoring (IOM). Univ Baltimore Law Rev.

